# One-Step Synthesis of Polymeric Carbon Nitride Films for Photoelectrochemical Applications

**DOI:** 10.3390/nano15130960

**Published:** 2025-06-21

**Authors:** Alberto Gasparotto, Davide Barreca, Chiara Maccato, Ermanno Pierobon, Gian Andrea Rizzi

**Affiliations:** 1 Department of Chemical Sciences, Padova University and INSTM, 35131 Padova, Italy; chiara.maccato@unipd.it (C.M.); ermanno.pierobon@unipd.it (E.P.); 2 CNR-ICMATE and INSTM, Department of Chemical Sciences, Padova University, 35131 Padova, Italy; davide.barreca@unipd.it

**Keywords:** polymeric carbon nitride films, in situ synthesis, chemical vapor infiltration, Ni foam, oxygen evolution reaction, water splitting

## Abstract

Over the last decade, polymeric carbon nitrides (PCNs) have received exponentially growing attention as metal-free photocatalytic platforms for green energy generation and environmental remediation. Although PCNs can be easily synthesized from abundant precursors in a powdered form, progress in the field of photoelectrochemical applications requires effective methods for the fabrication of PCN films endowed with suitable mechanical stability and modular chemico-physical properties. In this context, as a proof-of-concept, we report herein on a simple and versatile chemical vapor infiltration (CVI) strategy for one-step PCN growth on porous Ni foam substrates, starting from melamine as a precursor compound. Interestingly, tailoring the reaction temperature enabled to control the condensation degree of PCN films from melem/melon hybrids to melon-like materials, whereas the use of different precursor amounts directly affected the mass and morphology of the obtained deposits. Altogether, such features had a remarkable influence on PCN electrochemical performances towards the oxygen evolution reaction (OER), yielding, for the best performing systems, Tafel slopes as low as ≈65 mV/dec and photocurrent density values of ≈1 mA/cm^2^ at 1.6 V vs. the reversible hydrogen electrode (RHE).

## 1. Introduction

Polymeric carbon nitrides (PCNs), featuring a pseudo-graphitic structure and comprising C, N, and H (due to residual amino-groups) are a class of attractive and extremely versatile multi-functional materials for a variety of end-uses [1,2,3]. Among them, melon or melon-like systems, formed by variously condensed 1D chains of amine-linked heptazine units forming H-bonded layers, are the subject of numerous on-going research activities [2,4,5,6]. These poly-heptazines, often identified with the misnomer ‘graphitic carbon nitride’ [5,6,7], are formed by nontoxic abundant elements, can be synthesized from inexpensive precursors, and exhibit a high chemical/thermal stability and appealing characteristics. More specifically, melon-like PCN has a band-gap of ≈2.7 eV, suitable for Vis light absorption, and possesses band edge positions capable of promoting various reactions, such as O_2_ and H_2_ evolution, CO_2_ reduction, and the degradation of organic pollutants [7,8,9,10,11]. Interestingly, PCN reactivity and optoelectronic properties can be tailored by modulating its condensation degree and type/content of defects [2,11,12]. The control of such features, as well as of particle size and morphology [13,14], also allows to circumvent the intrinsic shortcomings of PCNs, including the limited surface area, low electric conductivity, and rapid recombination of photogenerated charge carriers [3,5,8,9].

PCNs are typically synthesized in a powdered form through the thermal polymerization of C,N-containing precursors, such as melamine, urea, or dicyandiamide [3,5,7,15]. Upon annealing in air or inert atmosphere, such compounds undergo a cascade of condensation/de-amination reactions yielding the initial formation of melam, and the subsequent generation of melem (and its oligomers, see below), that then evolves to melon-like PCN [4,5,16]. The modulation of polymerization degree [4,9,17] and amino groups concentration [1,7,18] provides a versatile toolbox for the molecular-level engineering of material reactivity and functional behavior. In this regard, recent works have reported that the photocatalytic performances of melem, its oligomers, and melem/melon hybrids might be superior to melon itself (the archetype PCN material), due to enhanced reactivity and lower electron/hole recombination [4,8,9,10,17]. Nonetheless, tailoring of PCN surface chemistry and defect content might also favorably impact on wetting properties, a remarkable issue for various (photo)catalytic/(photo)electrochemical reactions [19,20,21,22].

Although the synthesis of PCN powders is quite straightforward, the fabrication of films featuring good adhesion to conductive substrates, as required for photoelectrochemical applications, is more challenging and still in the early stages of development due to the difficulties in achieving adequate chemical, physical, and catalytic properties [22,23,24,25,26]. The most common approach to prepare PCN-based films involves the preliminary synthesis of PCN powders and, subsequently, their immobilization on substrates using methods such as spin-coating, dip/drop-casting, doctor-blade, or electrophoretic deposition [15,17,27,28]. Nevertheless, such approaches generally lead to weakly crystalline, inhomogeneous, and scarcely adherent deposits, featuring poor mechanical stability and modest electron transport capacity [12,18,23,27,29,30]. A direct, in situ polymerization route for the one-step growth of PCN films could overcome the above-mentioned problems, also avoiding the drawbacks related to powder processing methods [22,24,30,31,32]. Approaches for the direct synthesis of PCN films, much less investigated than the two-step routes employed for immobilizing PCN powders, include thermal vapor condensation, microcontact printing, solvothermal routes, electrochemical methods, and chemical vapor deposition (CVD). Up to date, such approaches have been mainly employed for PCN growth on 2D planar substrates, such as fluorine-doped tin oxide (FTO) or indium tin oxide (ITO)-coated glass [11,12,18,22,23,27,29,31,33,34,35], whereas reports on non-conventional porous supports are almost absent [14,30].

As a step forward in this general context, the present proof-of-concept investigation focuses on the in situ synthesis of PCN films on Ni foam supports through a chemical vapor infiltration (CVI) route. The developed strategy benefits from the molecular-level flexibility and infiltration power of CVD-related techniques to achieve the efficient dispersion of PCN into the porous substrate [12,14,18,36]. Modulation of preparative conditions enabled to tailor PCN loading and morphology, as well as its composition from melem/melon hybrids to melon-like systems. The resulting electrode architectures benefit from the optimal PCN/substrate mechanical adhesion and intimate interfacial contact [15,37], as well as from the high porosity and electrical conductivity of the Ni foam substrate [13,38,39], yielding promising catalytic performances towards the oxygen evolution reaction (OER), the bottleneck of water splitting [28,40]. To the best of our knowledge, no literature works have been reported to date on the OER performances of PCN-based photoelectrodes with different polymerization degrees.

## 2. Materials and Methods

### 2.1. Synthesis of Electrode Materials

In the present work, PCN was grown on Ni foam substrates (lateral size ≈1 × 2 cm^2^; thickness = 1.7 mm; Ni-4753, RECEMAT BV, Dodewaard, The Netherlands). For complementary chemico-physical and electrochemical characterization, depositions were also carried out on FTO-coated glass supports (lateral size ≈1 × 2 cm^2^; overall and FTO layer thickness = 2.2 mm and ≈600 nm, respectively; ≈7 Ω/sq; 735167-1EA, Sigma-Aldrich, Darmstadt, Germany), revealing the facile transferability of the proposed method to diverse support materials. Prior to each deposition, the substrates were subjected to an optimized cleaning procedure [32,41] and fixed on the top of a V-shaped alumina crucible, whose bottom had been previously filled with 100, 200, or 300 mg of pre-grinded melamine powders (99%, Sigma-Aldrich, Darmstadt, Germany). The crucible was hence positioned on a stainless steel susceptor, covered with a second larger alumina vessel, and introduced into a tubular furnace (Carbolite, HST 12/200, Derbyshire, UK) equipped with a quartz tube (inner diameter ≈ 9.5 cm; length of the heated region ≈ 20 cm). PCN deposition (duration = 2.5 h) was carried out at atmospheric pressure under flowing Ar (rate = 3 L/min) in the absence of additional co-reactants, at 500, 550, or 600 °C (heating rate = 5 °C/min). At the end of each experiment, samples were cooled down to room temperature under flowing Ar. The mass of each deposit was measured using a Mettler Toledo (Greifensee, Switzerland) XS105 DualRange microbalance.

### 2.2. Characterization of Electrode Materials

X-ray diffraction (XRD) measurements were performed in a glancing incidence configuration (θ_i_ = 1.0°) using a Bruker (Karlsruhe, Germany) AXS D8 Advance Plus diffractometer, equipped with a CuKα X-ray source (λ = 1.54051 Å). The average crystal size was estimated using the Scherrer equation. Optical absorption spectra were collected on a Cary 5E (Varian, Palo Alto, CA, USA) spectrophotometer (spectral bandwidth = 1 nm), operating in transmittance mode on FTO-supported materials. Band-gap (*E*_G_) values were estimated from the corresponding Tauc plots [(αhν)^1/2^ vs. hν], assuming indirect allowed transitions [28,42,43]. Field emission-scanning electron microscopy (FE-SEM) analyses were performed by collecting secondary (SE) and backscattered electron (BSE) signals using a Zeiss (Oberkochen, Germany) SUPRA 40VP instrument, at primary beam acceleration voltages of 10–20 kV. X-ray photoelectron spectroscopy (XPS) analyses were performed using a ThermoFisher (Waltham, MA, USA) ESCALAB 250XI+ apparatus, with a monochromatized AlKα X-ray source (hν = 1486.6 eV). Binding energy (BE) values were corrected for charging by setting the adventitious C1s component at 284.8 eV [44]. After a Shirley-type background subtraction, curve fitting was carried out using the XPSpeak 4.1 software [45], using Gaussian–Lorentzian sum functions. Atomic percentages (at.%) were calculated through peak area integration.

### 2.3. Functional Tests

OER electrochemical tests were carried out both in the dark and under irradiation, using an Autolab PGSTAT204 potentiostat/galvanostat workstation. The prepared samples, a Pt coil, and a Hg/HgO (MMO) electrode were used as working, counter-, and reference electrodes, respectively. The electrolyte was a 0.1 M KOH (Sigma-Aldrich, Darmstadt, Germany) aqueous solution (pH = 12.9). For measurements under irradiation, the working electrode was exposed to a white light LED source (Philips LUMILEDS LXML-PWN1 0120; intensity ≈100 mW/cm^2^). Potential values vs. MMO (E_MMO_) were converted into the reversible hydrogen electrode (RHE) scale using the following equation: E_RHE_(V) = E_WE_(V) + E_MMO_(V) + 0.0592 × pH, where E_WE_ indicates the bias applied to the working electrode. Linear sweep voltammetry (LSV) curves were recorded with a scan rate of 1 mV/s, after activating the samples with cyclic voltammetry (CV) measurements until constant behavior. To assess the material stability, chronoamperometric (CA) analyses were performed at a fixed potential value of 1.5 V vs. RHE under visible light irradiation. Experimentally measured currents were normalized to the electrode geometric area (≈2 cm^2^) [46], obtaining current densities in the dark (j_dark_), under irradiation (j_light_), and the corresponding photocurrent density values (j_light_ − j_dark_) [47]. Tafel slopes were determined by analyzing the plots of potential vs. log(current density). Electrochemical impedance spectroscopy (EIS) measurements were collected between 50 kHz and 0.5 Hz, with 5 mV sine perturbation.

During the execution of electrochemical experiments, Ni foam-supported specimens featured a marked capillary effect, evidenced by the rise of the electrolytic solution well above the sample immersion level. Because of this phenomenon, likely arising from both the substrate porous structure and the presence of polar groups in the obtained PCN deposits (see below), careful sealing of the electrode contact with epoxy resin turned out to be necessary to ensure a reproducible and accurate measurement setup [19,20,48].

The “coumarin test”, a simple and highly sensitive method to assess the relative activity of different catalysts [49], was used to probe the eventual production of •OH radicals by monitoring the formation of the highly photoluminescent species 7-hydroxy-coumarin [49,50,51]. To this aim, electrochemical experiments were carried out under illumination and constant stirring in a 0.1 M phosphate buffer solution (PBS, pH = 6.9) containing 1 mM coumarin (Sigma-Aldrich, Darmstadt, Germany), using the above-described setup and a saturated calomel reference electrode. Then, Ni foam-supported samples were tested at a fixed bias of 1.6 V vs. RHE, withdrawing, at regular time intervals, aliquots of the solution. The latter were analyzed by collecting fluorescence spectra in the range 340–700 nm using a FLS 1000 fluorimeter (Edinburgh Instruments, Livingston, UK) and adopting the following settings: excitation wavelength/bandwidth = 330/1.5 nm; emission bandwidth = 2.5 nm; optical path = 1 cm.

## 3. Results and Discussion

**Figure 1:**
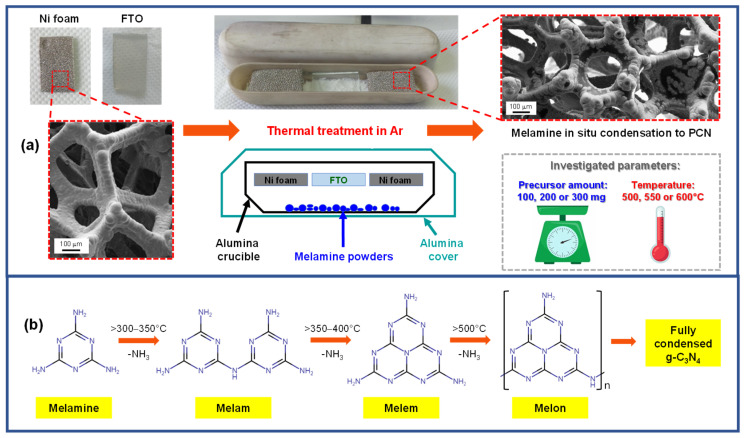
(**a**) Schematic representation of the setup and main process parameters used for PCN deposition on Ni foam and FTO substrates. (**b**) General sketch of melamine main condensation products as a function of temperature. Full condensation should lead to the idealized graphitic carbon nitride (g-C_3_N_4_) structure [4,54].

A sketch of the setup employed for the one-step growth of PCN deposits on Ni foam supports, along with the main synthesis parameters, is reported in Figure 1a. In a typical experiment, weighted amounts of finely grinded melamine powders (100, 200, or 300 mg) were uniformly spread on the bottom of a V-shaped alumina crucible. Subsequently, pre-cleaned Ni foam and FTO substrates of a suitable size were fit in the upper part of the crucible, ca. 1 cm above the precursor. A second larger crucible was hence positioned face-down on the former one to set up a semi-closed vessel, an important issue to minimize undesired precursor losses and ensure an adequate melamine delivery to the growth surface during the heating stage [52,53]. In this regard, the proper positioning of the top crucible over the bottom one also turned out to be critical to obtain PCN deposits with a reproducible mass. Finally, the double crucible system was positioned on a stainless steel susceptor, introduced into a tubular oven, and heated at the desired temperature in an Ar atmosphere. Under these conditions, precursor vapors infiltrate into the porous Ni foam, and the process is accompanied by de-amination and condensation reactions that yield different products depending on the adopted operating conditions. In this regard, Figure 1b provides a simplified scheme [4,12], reporting approximate temperatures for the progressive conversion of melamine into melam, melem, and melon-based species.

Nevertheless, it is worthwhile noticing that different compounds might coexist under certain conditions (e.g., melamine/melem or melem/melon adducts), and various oligomeric species (such as melem dimer, trimer, etc.) can also be formed depending on reaction temperature, time, and atmosphere. In this regard, a more detailed reaction path based on recent literature reports [4,54] is provided in Figure S1.

The composition of precursor residues at the end of the thermal treatment procedure was investigated via Fourier transform infrared (FT-IR) analyses. The corresponding spectra (Figure S2) revealed a broad band between 3000 and 3400 cm^−1^ resulting from the presence of uncondensed amino groups (NH_x_, x = 1,2) on PCN ring edges [55,56], beside adsorbed water arising from atmospheric exposure [56,57]. In the 1100–1800 cm^−1^ region, powders resulting from treatments at 550 and 600 °C revealed very similar spectral features, with peaks at 1638, 1568, 1416, 1316, and 1242 cm^−1^ well-matching the stretching modes of C=N/C-N heterocycles of a melon-type PCN material [55,58,59]. The signals at 886 cm^−1^ and 808 cm^−1^ are due to N-H deformation modes and to the breathing of triazine/heptazine units, respectively [13,55,56].

Regarding powders calcined at 500 °C, although the spectrum between 1100 and 1800 cm^−1^ resembled those collected on the 550 and 600 °C samples, the appearance of peaks at 1616, 1466, and 1330 cm^−1^ was consistent with the formation of melem oligomers [4,6,17,59]. Such findings suggest the formation of a melem/melon hybrid material, consistently with a lower melamine condensation degree at 500 °C.

A similar evolution as a function of temperature was also observed for Ni foam- and FTO-supported samples (vide infra). Additionally, tailoring of melamine amount in the reaction vessel (100, 200, or 300 mg) yielded deposits whose mass increased from (1.0 ± 0.2) mg to (4.0 ± 0.3) mg, a variation accompanied by an appreciable evolution of the corresponding material morphology (see below).

**Figure 2:**
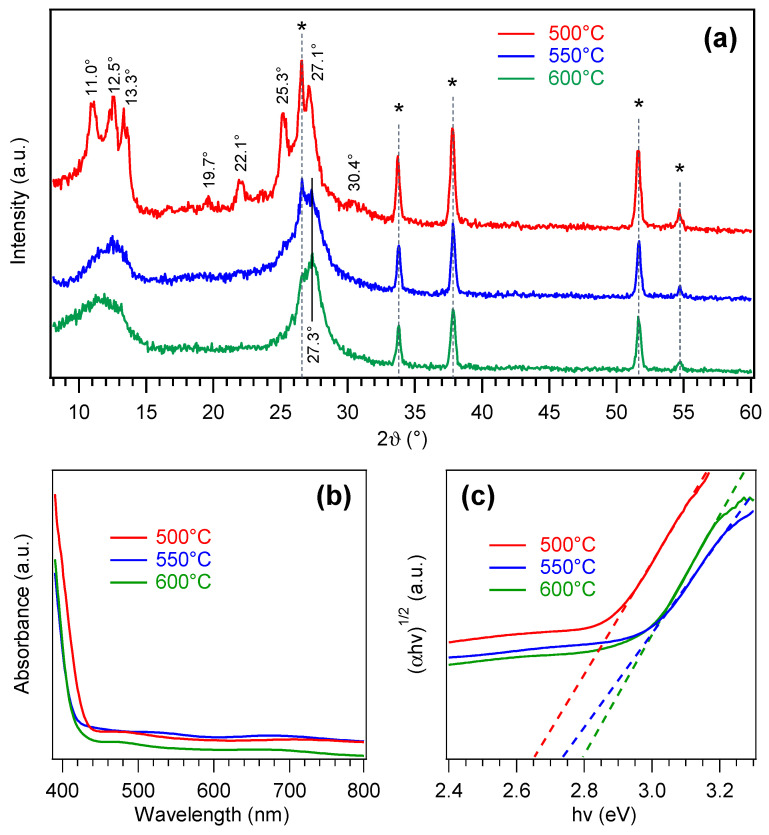
(**a**) XRD patterns of PCN films grown on FTO at different temperatures. Reflections labeled with * are due to the substrate. (**b**) Optical absorption spectra and (**c**) corresponding Tauc plots for the same specimens.

Figure 2a reports the XRD patterns of PCN films grown on FTO at different temperatures. In addition to peaks due to the substrate, the sample obtained at 500 °C exhibited reflections at 2ϑ ≈ 11.0, 12.5, 13.3, 19.7, 22.1, 25.3, 27.1, and 30.4°, which could be assigned to melem oligomers [4,9,60,61]. Nevertheless, the broad bands in the 10–15° and 25–30° regions suggested the co-presence of melon [5,6,53,62], as further supported by XPS results (see below).

At reaction temperatures of 550 and 600 °C, XRD patterns (relatively similar but markedly different from the 500 °C one) revealed two broad reflections at ≈12.0 and ≈27.0° that, together with the disappearance of several features detected for the 500 °C-grown sample, supported the occurrence of a melon-type material, in tune with a more extensive melamine condensation under harsher temperature conditions [3,5,62]. In particular, peaks at 2ϑ ≈ 12.0 and ≈27.0° can be attributed to melon periodicity along (100) crystallographic planes and (002) interplanar stacking, respectively [5,62,63]. The average crystallite sizes were estimated to be ≈3 nm, for samples grown at 550 and 600 °C, and ≈10 nm, for the specimen synthesized at 500 °C.

The above findings were consistent with the outcomes of optical absorption analysis (Figure 2b). Whereas the 500 °C sample presented an absorption onset at λ ≈ 440 nm, the other two systems featured a blue shift of ≈20 nm. Band-gap determination from Tauc plots (Figure 2c) yielded *E*_G_ ≈ 2.65 eV at 500 °C and ≈2.75–2.80 eV at 550 and 600 °C. Whereas for melon-like materials, *E*_G_ values lower than the ones for less-condensed PCN species are typically reported [4,17,53,59], the opposite trend was detected in the present case. Such a finding was traced back to the low nanocrystal size of samples fabricated at the higher temperatures, likely resulting in quantum confinement effects [14,28,30,63].

**Figure 3:**
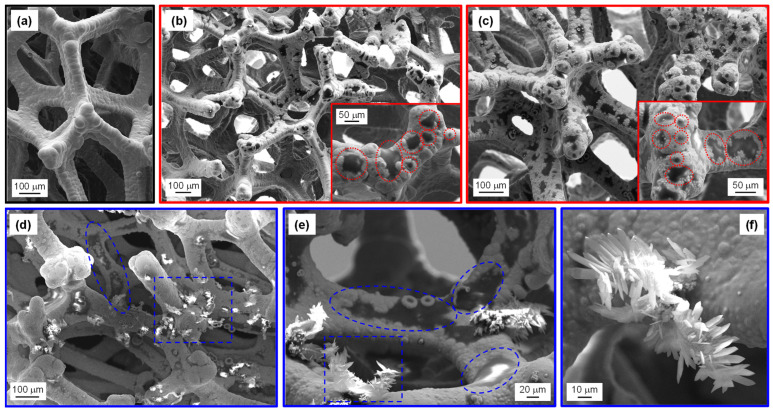
FE-SEM micrographs obtained from secondary electron (SE) signal for: (**a**) bare Ni foam; two representative samples grown at 500 °C from (**b**) 100 mg and (**c**) 300 mg of melamine. In the insets, some island-like PCN particles are highlighted by red circles; (**d**–**f**) different magnification images pertaining to a specimen grown at 550 °C from 300 mg of precursor. In panels (**d**,**e**), selected regions where island- and flake-like PCN structures appear well-evident are enclosed by blue circles and rectangles, respectively.

FE-SEM analyses for Ni foam-supported samples are reported in Figure 3, Figures S3 and S4. The bare Ni foam (Figure 3a) was characterized by a highly porous 3D structure consisting of interconnected branches with a diameter of ≈100 μm. Upon CVI at 500 °C, the Ni foam presented dark regions ascribed to the presence of PCN particles, as revealed by SE micrographs in Figure 3b,c. In this regard, the atomic mass-sensitive BSE image in Figure S3 provides more effective evidence for PCN distribution (revealed by dark-contrast regions) over the Ni foam (bright-contrast). In particular, BSE imaging revealed that even areas where PCN was apparently absent according to the corresponding SE micrograph could be covered by PCN. In this regard, it is worth highlighting that the characterization of Ni foam-supported materials via electron microscopy and spectroscopic techniques might be challenging due to the system multi-scale morphological complexity and tortuous internal structure, limiting collection of emitted electrons to a narrow range of takeoff angles [39,64].

Basing on the obtained data, specimens synthesized at 500 °C were characterized by PCN particles with an island-like morphology, well-adherent to the underlying Ni foam, an important goal for electrochemical applications [22,23]. A similar morphology has been reported for CVD-grown carbon nitride films on conventional planar substrates [33]. The extent of substrate coverage by PCN islands increased with the precursor amount used during the CVI process (compare Figure 3b,c). Such a result was in line with the different masses of the corresponding deposits (see above).

PCN “islands” were revealed by FE-SEM measurements even for samples fabricated at 550 and 600 °C. Nevertheless, whereas such particles were the only detectable when the synthesis was carried out from 100 mg of precursor, the presence of agglomerated lamellar structures (typical thickness and length ≈ 1–2 and ≈20 μm, respectively) was also noticed when melamine amount was increased, in particular, to 300 mg (see Figure 3d–f). The formation of such “aggregates” (see also Figure S4) at higher reaction temperatures suggested the occurrence of a different growth mechanism under harsher processing conditions. Although the high surface-to-volume ratio of flake-like PCN might appear favorable for the target application, preliminary OER experiments evidenced that such lamellar structures yielded unstable electrochemical performances upon prolonged utilization. Based on the comparison of FE-SEM micrographs collected prior and after functional tests (compare Figure 3d–f with Figure S12a), this effect was traced back to a partial detachment of PCN flakes from the Ni foam substrate. Such a phenomenon, also responsible for the clouding of the electrolytic solution, was particularly critical for specimens synthesized at 600 °C.

The composition of representative samples grown at 500 and 550 °C was investigated by XPS. Survey spectra and surface atomic percentages (see Figure S5 and Table S1) revealed the presence of photoelectron and Auger signals from nitrogen and carbon, along with oxygen and nickel in lower amounts [44,65]. Detection of the latter suggested partial exposure of the Ni foam substrate, in line with FE-SEM results.

**Figure 4:**
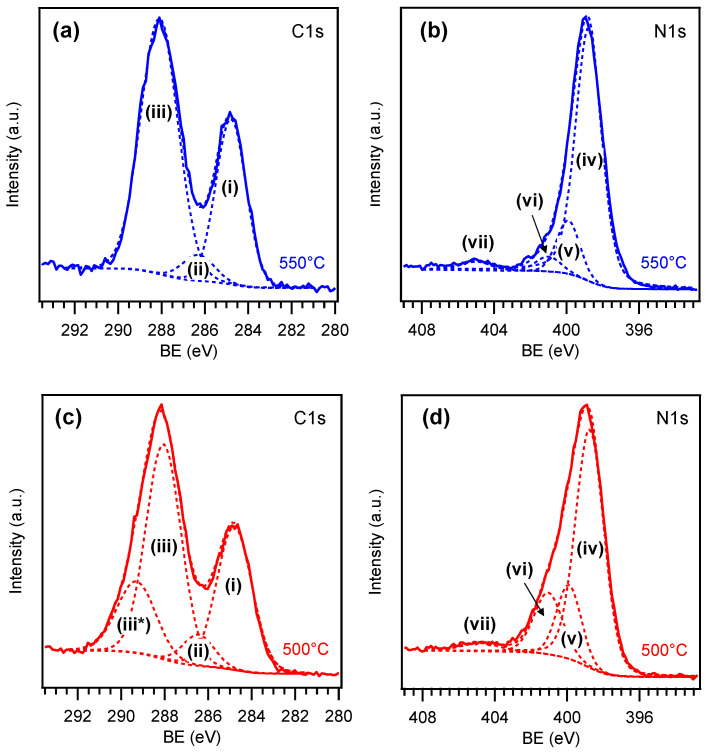
C1s and N1s XPS signals pertaining to two PCN samples grown on Ni foam at (**a**,**b**) 550 and (**c**,**d**) 500 °C.

Important information was gained from the analysis of C1s and N1s photopeaks in Figure 4, revealing a broadening of both signals on the high BE side for the sample synthesized at 500 °C in comparison to the homologous one obtained at 550 °C. Concerning the latter sample, the C1s peak could be deconvoluted into three components (Figure 4a). Band (i), centered at 284.8 eV, was traced back to adventitious carbon contamination [44,65]. A second relatively weak contribution [(ii); BE = 286.3 eV] was attributed to carbon atoms bonded to uncondensed amino groups (C-NH_x_, with x = 1, 2) on heptazine ring edges. The third major band (iii) at 288.1 eV was ascribed to N-C=N carbon atoms in the PCN network [6,14,28,53,66]. For the N1s signal of the 550 °C-grown sample, four contributing components were identified (Figure 4b). The main one [(iv); BE = 398.8 eV] was traced back to bi-coordinated N atoms of PCN moieties (C=N-C, N_2c_), whereas band (v) at 399.9 eV was attributed to tertiary N centers [N-(C)_3_, N_3c_] [6,28,40,53,66,67]. Peak (vi) at 401.0 eV was consistent with -NH_x_ presence, and peak (vii), at 404.9 eV, was attributed to the excitation of π electrons [28,40,66,68,69].

The C1s and N1s peaks for the sample synthesized at 500 °C are reported in Figure 4c and 4d, respectively. For the former signal, the assignment of components (i), (ii), and (iii) is the same reported above. The additional band (iii*) at 289.3 eV was ascribed to oxidized carbon species involving C-O/C=O moieties on PCN surface [8,70,71]. The N1s peak (Figure 4d), deconvoluted as the one plotted in Figure 4b, revealed a larger relative contribution of band (vi) to the overall signal, in tune with a higher content of uncondensed amino groups at 500 °C. Accordingly, the N/C ratio, calculated excluding the adventitious carbon contribution and based on peak fitting results in Table S2, was ≈1.5 for the specimen grown at 550 °C, in good agreement with the nominal value expected for a melon-like material [59]. Nonetheless, as far as the sample synthesized at 500 °C is concerned, an N/C ratio of ≈1.6 was obtained, supporting the formation of a less condensed melem/melon hybrid system [59]. Although subject of ongoing investigations, several theoretical and experimental studies attribute to edge amine nitrogen atoms a beneficial role in promoting several catalytic reactions, behaving as active centers and improving charge carriers separation, with amino groups acting as hole-stabilizers [60,72,73,74,75]. Additionally, together with –OH groups, –NH_x_ moieties also produce a more hydrophilic surface that facilitates electrolyte penetration into the foam porous structure, accelerates diffusion of hydroxyl-based reactants, and favors the removal of O_2_ bubbles, beneficially impacting on OER activity [19,20,22,72].

**Figure 5:**
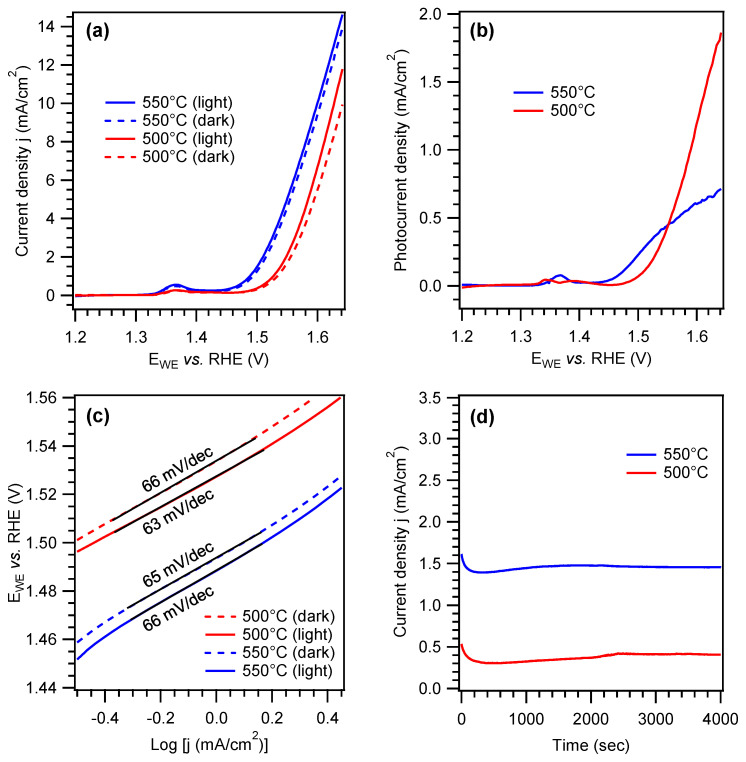
(**a**) LSV anodic scans in 0.1 M KOH collected in the dark (j_dark_, dotted lines) and under irradiation (j_light_, continuous lines) on two Ni foam-supported PCN samples grown at 500 °C and 550 °C from 100 and 200 mg of melamine, respectively. The weak anodic peak at ≈1.35 V vs. RHE is due to the NiO ⟶ NiO(OH) reaction involving the uncovered Ni foam surface [13,32,64]. (**b**) Photocurrent density values and (**c**) Tafel plots for the same specimens. In the latter case, continuous black lines mark the fitting of experimental curves. (**d**) Chronoamperometric curves collected under irradiation at 1.5 V vs. RHE.

The electrochemical performances of Ni foam-supported PCN samples were hence tested in 0.1 M aqueous KOH. Results pertaining to the characterization of samples grown at 500, 550, or 600 °C from different melamine amounts are provided in the Supporting Material (see Figures S7–S9 and pertaining discussion), while selected data for the best-performing specimens synthesized at 500 and 550 °C are reported in Figure 5. As a general rule, the samples featured an appreciable current density increase at potentials higher than ≈1.50 V vs. RHE (see LSV curves in Figure 5a and Figure S7a–c), underscoring their OER activity. For each specimen, current densities under irradiation were found to be higher than the corresponding values in the dark, confirming the photoactive nature of PCN deposits [13,15,35]. Analysis of onset potentials (Figure S8) revealed more favorable values for specimens synthesized at 550 and 600 °C, along with a moderate improvement under irradiation. Nonetheless, whereas samples grown at 500 °C were characterized by a retarded onset potential, as well as lower j_dark_ and j_light_ values compared to the systems synthesized at 550 and 600 °C (Figure S9a), they yielded the highest photocurrent density at sufficiently high bias values (see Figure 5b and Figure S9b), provided that an optimal PCN amount was loaded into the Ni foam. Overall, the above functional results are well placed among those reported for various PCN films fabricated either via powder immobilization or direct in situ growth [15,18,27,31,35,76], also taking into account that neither co-catalysts nor sacrificial agents were used in the present case (see also Table S3). The higher photocurrent produced around ≈1.50 V by the 550 °C-grown sample (Figure 5b) can be traced back to an enhanced thermal condensation degree [15,23,76]. Nonetheless, above ≈1.55 V vs. RHE, the sample deposited at 500 °C likely benefits from its superior light-harvesting properties (see above) [10,27].

Tafel slope values for the best-performing samples (≈65 mV/dec, Figure 5c), indicating promising reaction kinetics at the electrode surface, compared favorably with other PCN-based systems reported in the literature [24,40,70]. Under optimized deposition conditions, the proposed CVI approach yielded Ni foam-supported PCN samples with good operational stability, as revealed by the chronoamperometric measurements in Figure 5d. In this regard, FE-SEM analysis after OER experiments revealed that the island-like PCN structures detected prior to functional tests were still clearly evident, without any appreciable alteration (compare Figure 3c and Figure S12b), thus supporting the good morphological and electrochemical stability of PCN deposits and their effective adhesion to the Ni foam.

Overall, for the best-performing systems synthesized at 500 and 550 °C, current density values of 10 mA/cm^2^ were achieved under irradiation with an overpotential of ≈395 and ≈370 mV, respectively, whereas the corresponding photocurrents at 1.6 V vs. RHE were ≈1.2 and ≈0.6 mA/cm^2^ (Figure 5a,b). As a term of comparison, we have recently reported a two-step method involving: (i) the synthesis of PCN powders by thermal condensation of melamine in Ar atmosphere; (ii) the electrophoretic deposition of the resulting powders on Ni foam [13]. Although experimental conditions for melamine condensation were very similar to the ones adopted herein for the one-step PCN growth, OER performances were appreciably lower, with a photocurrent of ≈0.1 mA/cm^2^ at 1.6 V vs. RHE. Additionally, the NiO ⟶ NiO(OH) oxidation peak at 1.35–1.40 V vs. RHE was appreciably more evident, revealing an uneven PCN distribution on the substrate that negatively affected the system conductivity and produced a modest light response [13]. Conversely, the improved functional behavior of the present samples is likely due to the more uniform and effective dispersion of PCN particles into the substrate pores, resulting in an intimate PCN/Ni foam interfacial contact. Nonetheless, considering that PCN has shown an *n*-type behavior (see also Figure S11) and that appreciable current densities are observed only above the potential required for NiO(OH) formation, a co-operative mechanism between the Ni foam and PCN can be hypothesized under illumination, with holes being injected into the electrolyte through NiO(OH) sites, as well as through PCN. In fact, the oxidation potential for Ni^2+^/Ni^3+^ falls within the PCN energy gap. The ratio between these two paths should depend on the degree of coverage of the Ni foam.

Ni foam-supported samples were also investigated by EIS, both in the dark and under irradiation (see Figure S10a), revealing decreased charge transfer resistance in the latter case, in agreement with LSV curves in Figure 5a. As can be observed, EIS spectra were quite noisy. Such an effect is likely due to the strong O_2_ bubbling at high bias values and difficult charge redistribution due to shadowing effects arising from the substrate morphology, also resulting in a relatively modest light response. Mott–Schottky analysis (Figure S10b) revealed that the flat band potential was positioned at ≈1.4 V, in agreement with Figure 5a results.

To supplement (photo)electrochemical data, additional characterization experiments were carried out also on FTO-supported specimens, featuring a much faster light response. To this regard, LSV curves (under dark, light, and chopped conditions), as well as chopped light chronoamperometry and open circuit potential (OCP) scans, were collected (Figure S11). Overall, such data revealed an *n*-type behavior, instead of the amphoteric one often reported for polymeric carbon nitride [28,77]. Additionally, both chronoamperometric and OCP measurements supported the good material electrochemical stability. The slight photocurrent decrease observed in Figure S11b is due to hole accumulation on PCN surface (OCP shifts to higher values after application of a high anodic bias).

As far as the issue of mechanical stability is concerned, on these specimens, allowing to qualitatively assess PCN adhesion by visual inspection, no appreciable detachment/delamination was observed after performing the scotch tape test. As a matter of fact, the deposit could be removed from the substrate only by energy-intensive mechanical scratching.

**Figure 6:**
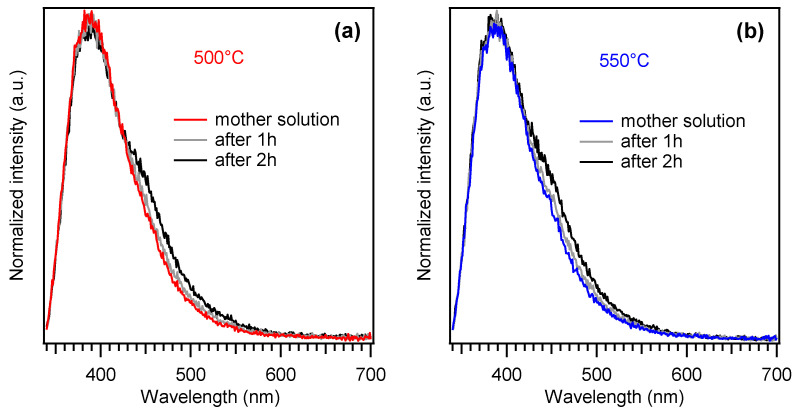
Luminescence spectra obtained from a 1 mM coumarin solution in 0.1 M phosphate buffer after 1 and 2 h of photoelectrochemical work under illumination at a fixed bias of 1.6 V vs. RHE. Panels (**a**,**b**) refer to results obtained on Nifoam-supported PCN samples grown at 500 °C and 550 °C from 100 and 200 mg of melamine, respectively.

Ni foam-supported specimens were finally investigated using the “coumarin test”, a benchmarked procedure to evaluate a catalyst’s ability to produce hydroxyl radicals (•OH) under irradiation. Whereas the formation of such species is highly desired for the degradation of organic aqueous pollutants, we have recently reported that the two-electron mechanism responsible for •OH generation might be competitive with the four-electron process involved in OER [13]. In this regard, results reported in Figure 6 clearly revealed a negligible formation of hydroxyl radicals by the present PCN-based materials, suggesting that such electrocatalysts feature a high selectivity towards water oxidation. In fact, the peak at ≈390 nm is due to coumarin emission, whereas the shoulder at ≈450 nm is due to 7-hydroxy-coumarin (7-OHC), whose formation takes place in the presence of photogenerated •OH [13,49]. Taking into account the much higher photoluminescence quantum yield of 7-OHC, the weak intensity of the corresponding signal reveals a negligible production of hydroxyl radicals.

## 4. Conclusions

In this work, we have proposed a cheap and versatile one-step fabrication route to PCN films with tunable compositional, structural, and optical properties. The proposed synthetic approach was optimized on Ni foam substrates yielding *n*-type PCN materials with good adhesion to the substrate, but can also be conveniently extended to other supports. The results of a multi-technique characterization by means of complementary analytical tools indicated that the simple tailoring of preparative conditions, such as reaction temperature and precursor amount, afforded the obtainment of PCN deposits with diversified morphological features and a tunable condensation degree (from melem/melon hybrids to melon-like materials). The modulation of such properties significantly impacted on the OER activity of the corresponding materials yielding, for the best-performing systems, photocurrent density values close to ≈1 mA/cm^2^ at 1.6 V vs. RHE and Tafel slopes as low as ≈65 mV/dec. Such results candidate the present materials as potentially promising catalysts for different electrochemical applications. In this regard, additional room for improvement can be provided by performance enhancement strategies, such as co-catalyst deposition, doping, or heterojunction engineering. Efforts in this direction are already under way.

## Data Availability

Data supporting this study are available within the article.

## Supplementary Materials

The following are available online at https://www.mdpi.com/article/10.3390/nano15130960/s1, Figure S1: additional details on the reaction mechanism; Figure S2: FT-IR spectra of powders obtained from melamine calcination; Figures S3 and S4: FE-SEM micrographs for representative samples grown at 500 and 550 °C, respectively; Figure S5: XPS survey spectra pertaining two PCN samples grown at 500 and 550 °C; Tables S1 and S2: XPS surface atomic percentages and breakdown of C1s and N1s atomic percentages according to peak fitting deconvolution for the same specimens; Figure S6: Ni2p and O1s XPS signals pertaining two samples grown at 550 and 500 °C; Figure S7: LSV anodic scans collected on Ni foam-supported PCN samples grown at 500 °C, 550 °C, and 600 °C, from different melamine amounts; Figure S8: onset potential under dark and light conditions for PCN samples in Figure S7; Figure S9: current density values in the dark and under irradiation, and photocurrent density values for Figure S7 specimens; Table S3: OER electrochemical performances of selected carbon nitride electrocatalysts reported in the literature; Figure S10: Nyquist plots for Ni foam-supported samples grown at 500 °C and 550 °C and representative Mott-Schottky plot; Figure S11: LSV curves in the dark and under illumination for a FTO-supported sample grown at 550 °C along with chronoamperometric and OCP scans under chopped light for the same material; Figure S12: FE-SEM micrographs recorded after electrochemical tests on Ni foam-supported samples grown at 550 °C and 500 °C. The authors have cited additional references in the Supplementary Materials [78,79,80,81,82,83,84,85].
