# Supplementary material for: One-Step Synthesis of Polymeric Carbon Nitride Films for Photoelectrochemical Applications
Source: Nanomaterials (Basel). 2025 Jun 21;15(13):960. doi: 10.3390/nano15130960 (PMC12250734; doi:10.3390/nano15130960)
Supplement: Supplementary file 1 [file nanomaterials-15-00960-s001.zip › nanomaterials-3659980-supplementary.pdf]

nanomaterials

Supporting Material.

One-Step Synthesis of Polymeric Carbon Nitride Films for Photoelectrochemical Applications

Alberto Gasparotto [1,2,*], Davide Barreca [2], Chiara Maccato [1,2], Ermanno Pierobon [1] and Gian Andrea Rizzi [1,2,*]

1 Department of Chemical Sciences, Padova University and INSTM, 35131 Padova, Italy; chiara.maccato@unipd.it (C.M.); ermanno.pierobon@unipd.it (E.P.)
2 CNR-ICMATE and INSTM, Department of Chemical Sciences, Padova University, 35131 Padova, Italy; davide.barreca@unipd.it
* Correspondence: alberto.gasparotto@unipd.it (A.G.); gianandrea.rizzi@unipd.it (G.A.R.); Tel.: +39-0498275192 (A.G.); +39-0498275722 (G.A.R.)

S1. Synthesis

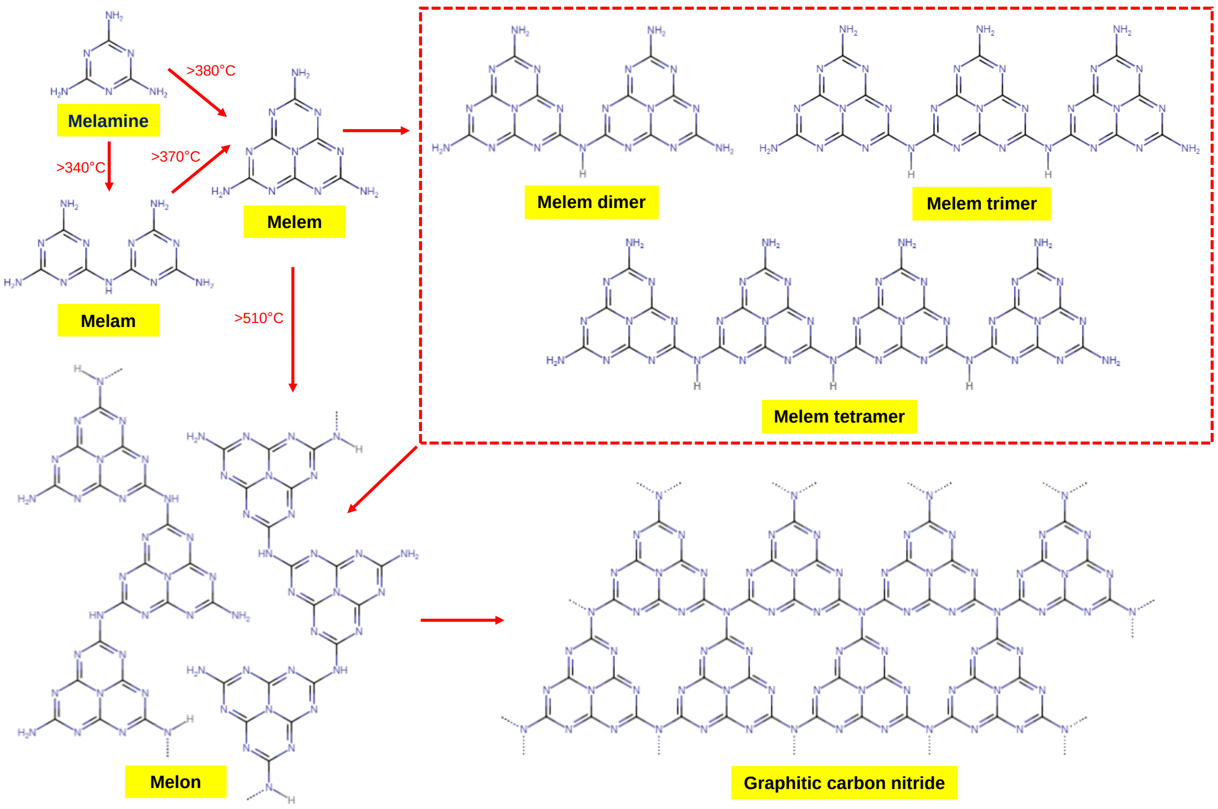

Figure S1. Proposed condensation route from melamine to melam, melem, and some possible oligomers, finally yielding the polymer melon. The structure of fully condensed graphitic carbon nitride is also reported. Scheme readapted from [1,2].

S2. Chemico-physical characterization

S2.1. Characterization of PCN powders

FT-IR analyses were carried out by preparing pellets of PCN powders in KBr and collecting transmittance spectra on a Thermo-Nicolet Nexus 860 instrument (resolution = 2 cm^{-1}).

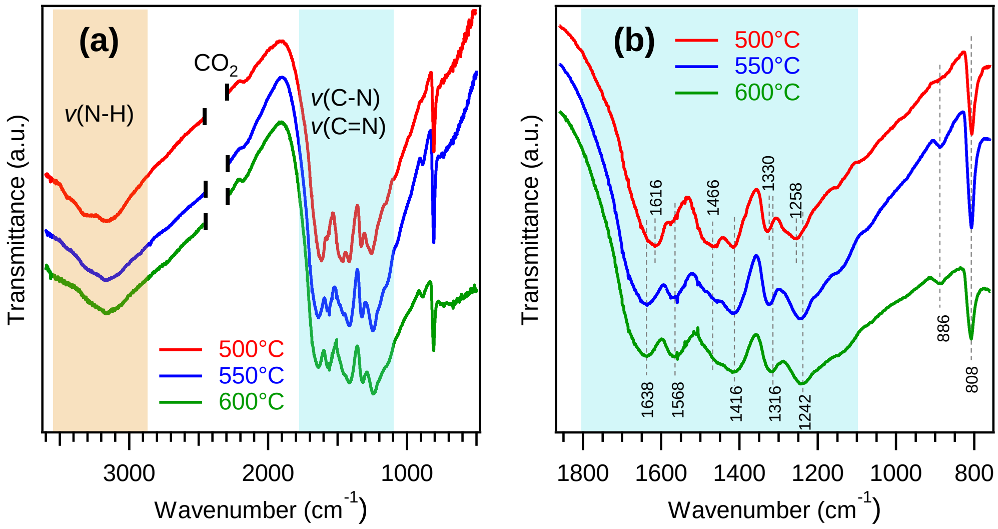

Figure S2. (a) FT-IR spectra of powders obtained from melamine calcination at different temperatures. The spectra have been vertically shifted for clarity. (b) Enlargement of the 1800-800 cm^{-1} region shown in (a).

S2.2. Chemico-physical characterization of Ni foam-supported PCN-based electrodes

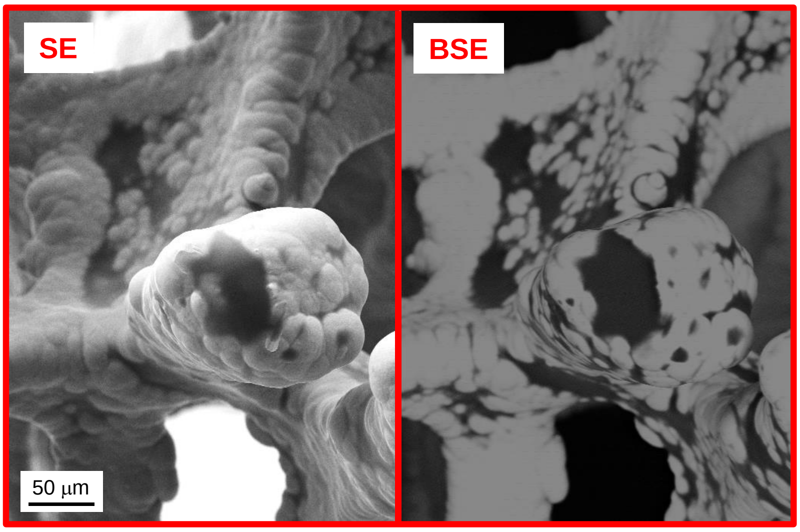

Figure S3. FE-SEM micrographs for a sample grown at 500°C from 100 mg of melamine. Left and right images were obtained collecting secondary electrons (SEs) and back-scattered (BSE) electrons, respectively. The PCN presence is more effectively evidenced by dark regions in the right-panel image.

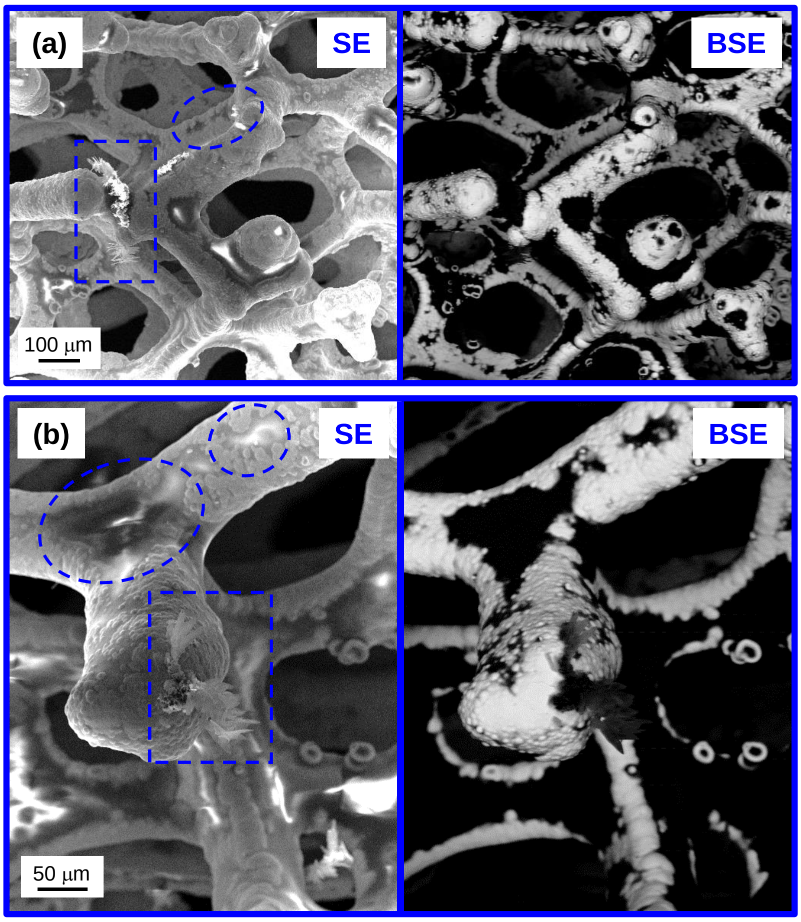

Figure S4. (a-b) FE-SEM micrographs for a specimen grown at 550°C from 200 mg of precursor and acquired by collecting SE (left) and BSE (right) signals, respectively. Some representative regions where island- and flake-like PCN structures are more evident are enclosed by blue circles and rectangles, respectively, in SE panels. Such structures appear as dark-contrast regions in BSE micrographs.

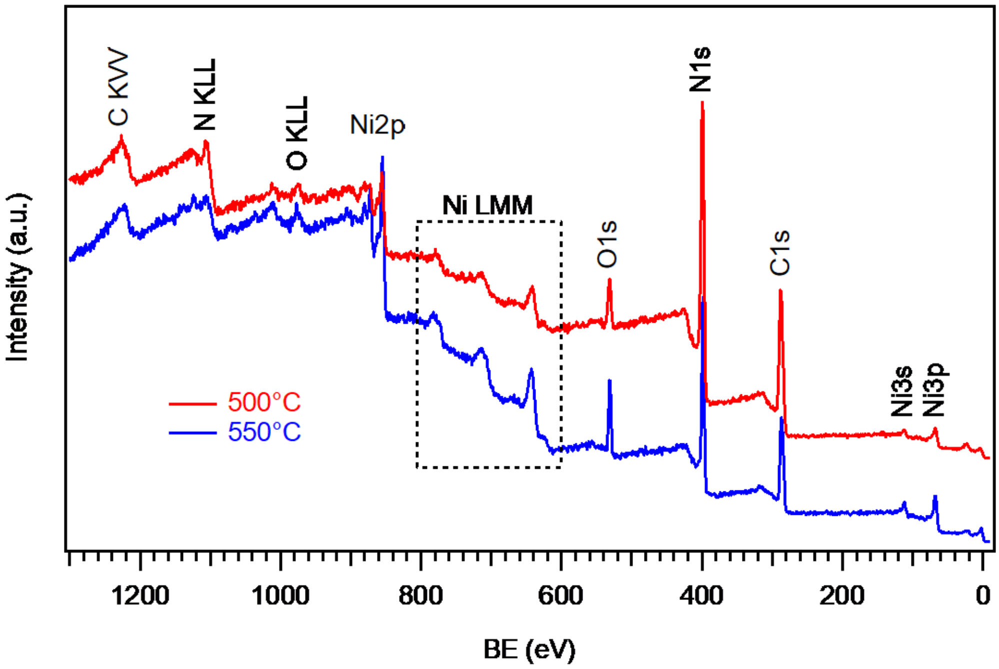

Figure S5. Wide-scan XPS spectra pertaining to two PCN samples grown on Ni foam at 500 and 550°, from 100 and 200 mg of precursor, respectively.

Table S1. XPS surface atomic percentages (at.%) for the two specimens reported in Figure S5.

	C1s (at.%)	N1s (at.%)	O1s (at.%)	Ni2p (at.%)
500°C	44.8	47.7	5.9	1.6
550°C	42.4	43.4	9.6	4.6

Table S2. Breakdown of C1s and N1s XPS atomic percentages (at.%) given in Table S1 according to the peak fitting deconvolution reported in Figure 4.

	C1s (at.%)	N1s (at.%)
**500°C**	**44.8**	**47.7**
	(i) = 14.0	(iv) = 30.6
	(ii) = 2.7	(v) = 7.8
	(iii) = 20.5	(vi) = 7.9
	(iii*) = 7.6	(vii) = 1.4
**550°C**	**42.4**	**43.4**
	(i) = 13.8	(iv) = 34.7
	(ii) = 2.2	(v) = 5.8
	(iii) = 26.4	(vi) = 1.7
		(vii) = 1.2

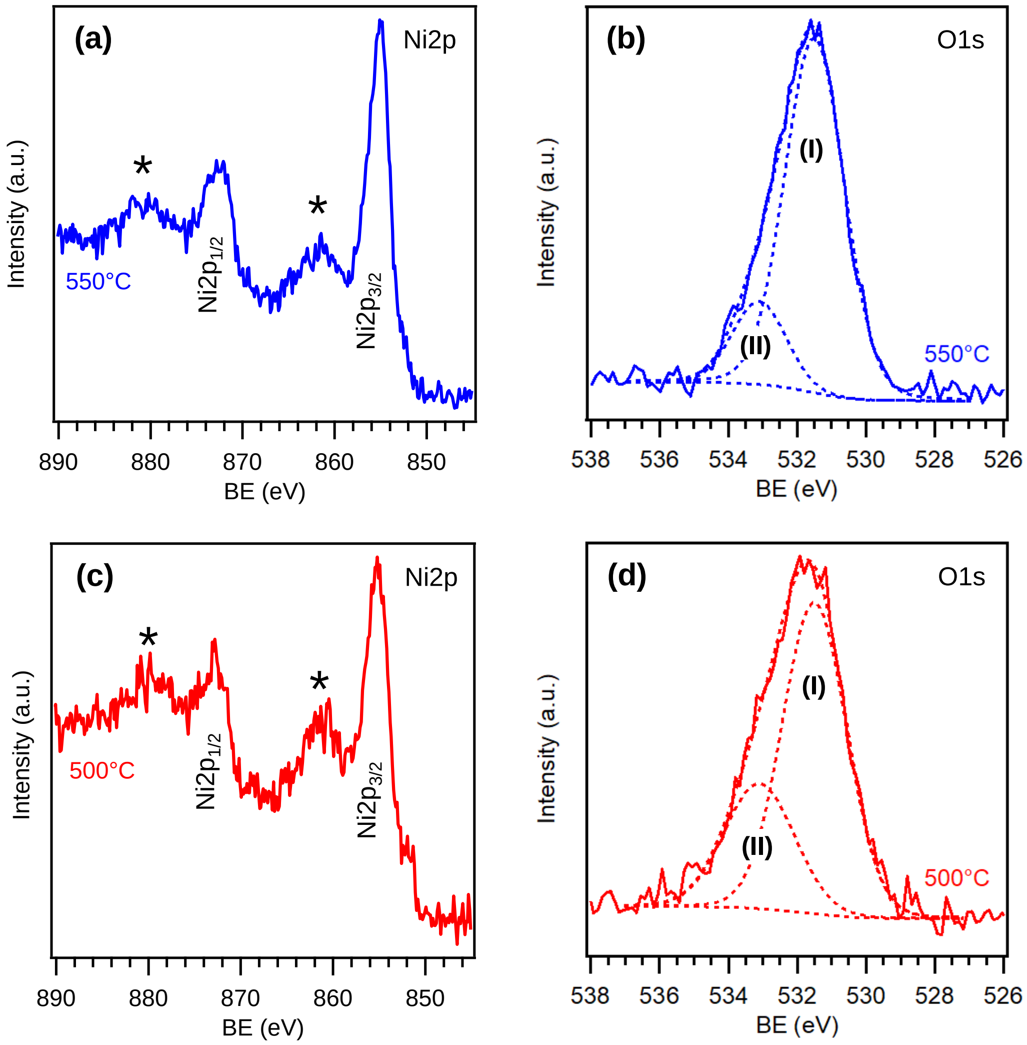

Figure S6. Ni2p and O1s XPS signals pertaining two PCN samples grown on Ni foam at (a-b) 550 and (c-d) 500°C. In panels (a,c), the symbol * indicates shake-up satellites [3,4]. In panels (b) and (d), fitting components labeled with (I) and (II) are due to hydroxyl groups and adsorbed water, respectively.

The Ni2p [BE($Ni2p_{3/2}$) ≈855.2 eV; spin-orbit-splitting (SOS) ≈17.5 eV] and O1s spectral features (Figure S6a-d) were consistent with the presence of a highly hydroxylated nickel foam surface [3,4]. In fact, deconvolution of the O1s signal revealed a main component at 531.5 eV ascribed to –OH groups, along with an additional weaker contribution at 533.2 eV traced back to molecularly adsorbed water [3,5]. It is likely that both –OH and H_2O species, of which their occurrence can be traced back to atmospheric exposure, are not only present on the uncovered substrate regions but also on PCN deposits.

S3. Functional tests of Ni foam-supported PCN-based electrodes

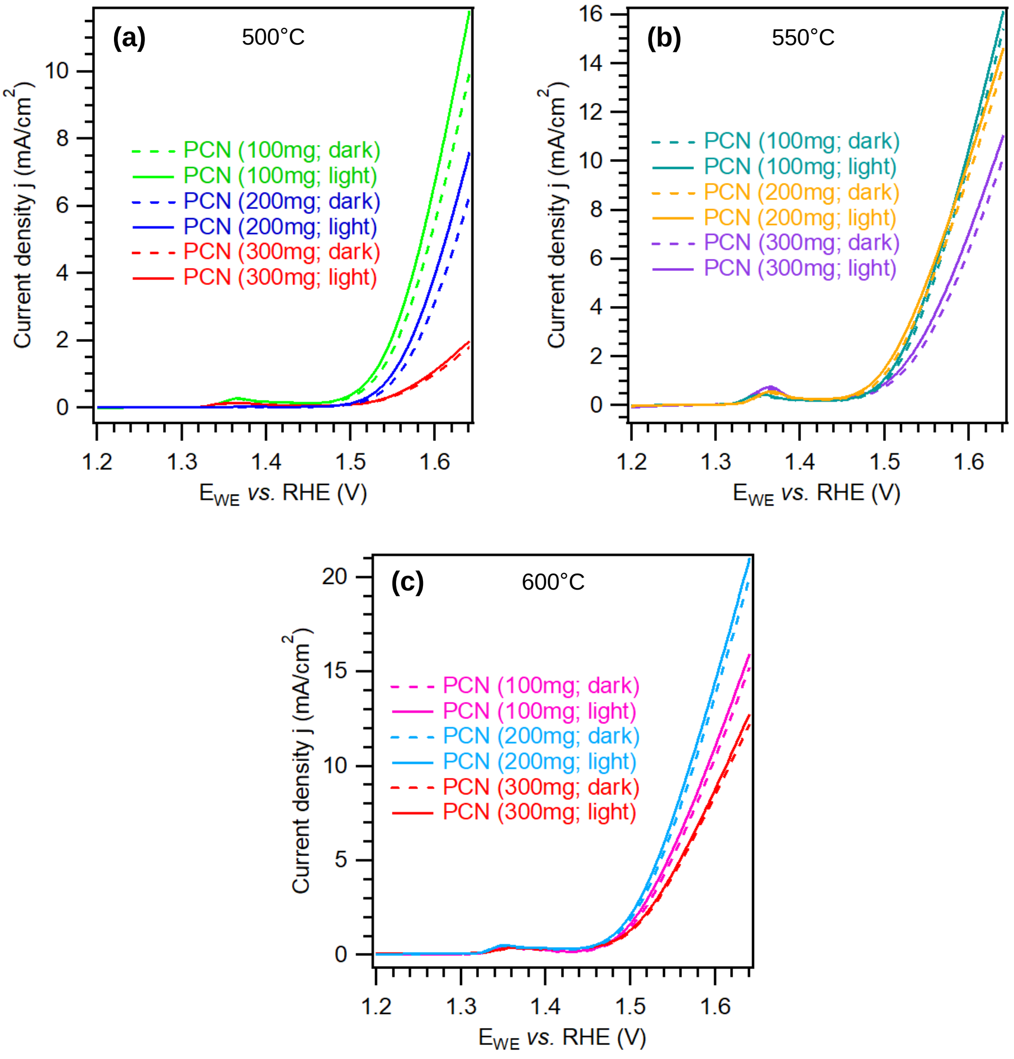

Figure S7. LSV anodic scans in 0.1 M KOH collected in the dark (j_{dark}, dotted lines) and under irradiation (j_{light}, continuous lines) on Ni foam-supported PCN samples grown at (a) 500°C, (b) 550°C, (c) 600°C, from different melamine amounts.

Whereas samples obtained at 600°C produced high j_{dark} and j_{light} values during the first LSV scans, they were found to be electrochemically unstable upon prolonged operation, an effect traced back to the partial detachment of flake-like PCN structures (see also Figure S12 and pertaining comment in the main paper). A similar phenomenon was also partially detected for samples synthesized from 300 mg of melamine at 500 and 550°C.

Compared to the best electrode materials discussed in the main paper, samples synthesized from 300 mg of precursor yielded higher Tafel slopes (≈85 mV/dec) irrespective of the growth temperature, indicating that too high of PCN loading results in the worsening of electrochemical performances.

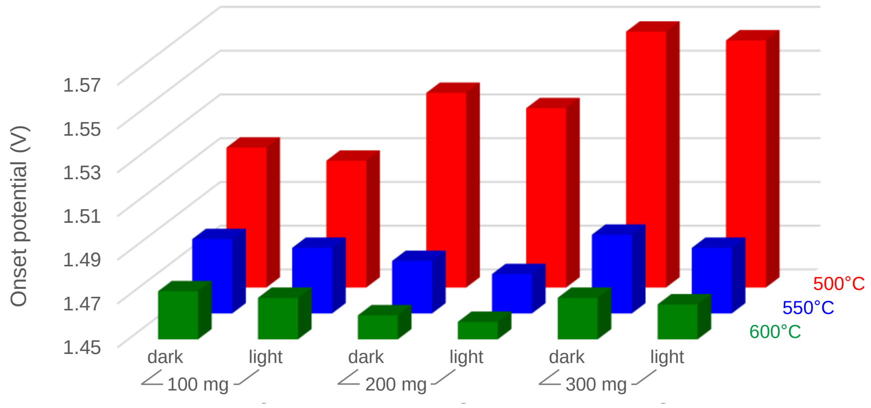

Figure S8. Onset potential under dark and light conditions for PCN samples in Figure S7a-c. The values were calculated from the corresponding LSV curves as the ones required to achieve a current density of 0.5 mA/cm^2.

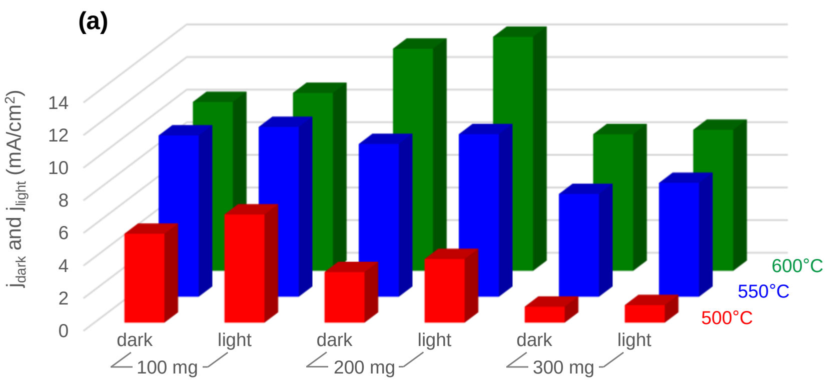

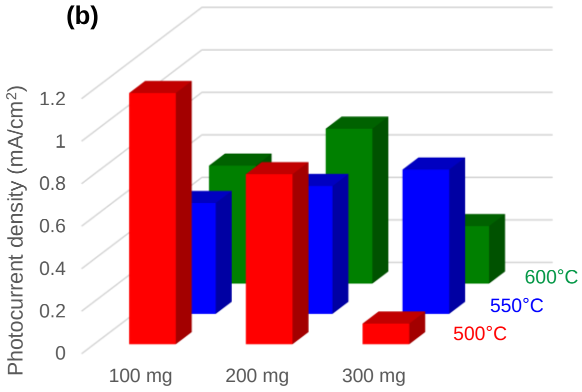

Figure S9. (a) Current density values in the dark (j_{dark}) and under irradiation (j_{light}) calculated at 1.6 V vs. RHE for PCN samples reported in Figure S7a-c. (b) Photocurrent density values (j_{light} - j_{dark}) for the same specimens.

Electrolyte	$j_{1.6}$ (mA/cm²)	Ref.
KOH 1.0 M	≈ 0.05 [a]	[6]
Na_2SO_4 0.1 M	≈ 0.14 [b]	[7]
Na_2SO_4 0.2 M	≈ 0.015 [b]	[8]
KOH 1.0 M	≈ 0.009 [b]	[9]
Na_2SO_4 0.5 M	≈ 0.009 [b]	[10]
NaOH 0.1 M	≈ 0.02 [b]	[11]
Na_2SO_4 0.1 M [c]	≈ 0.18 [b]	[12]
Na_2SO_4 0.2 M	≈ 0.004 [b]	[13]
Na_2SO_4 0.5 M	≈ 0.4 [b]	[14]
Na_2SO_4 0.1 M	≈ 0.06 [b]	[15]
Na_2SO_4 0.1 M	≈ 0.06 [a]	[16]
Na_2SO_4 0.1 M	≈ 0.1 [b]	[16]
Na_2SO_4 0.1 M	≈ 0.07 [b]	[17]
Na_2SO_4 0.5 M	≈ 0.005 [b]	[18]

Table S3. OER electrochemical performances of selected electrocatalysts based on bare carbon nitride. n.a. = not available. [a] dark conditions; [b] light conditions; [c] test carried out in the presence of sacrificial agents.

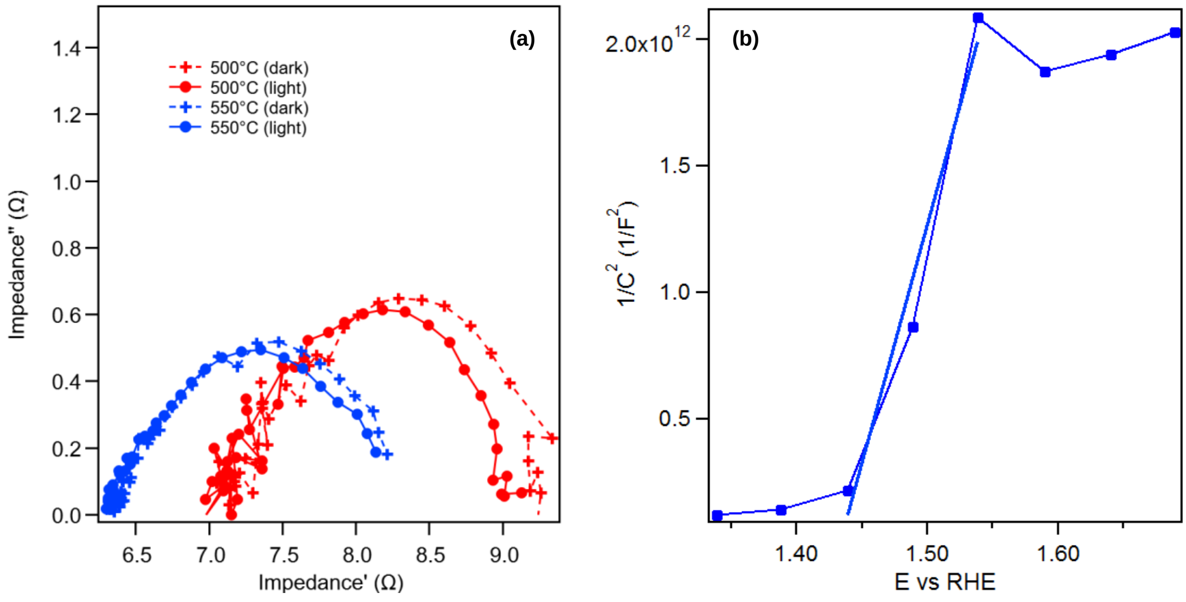

Figure S10. (a) EIS spectra, reported as Nyquist plots and collected at 1.65 V vs. RHE, for Ni foam-supported samples grown at 500°C and 550°C from 100 and 200 mg of melamine, respectively. (b) Representative Mott–Schottky plot obtained at 1000 Hz for the 550°C-grown sample reported in panel (a).

S4. Functional tests of FTO-supported PCN-based electrodes

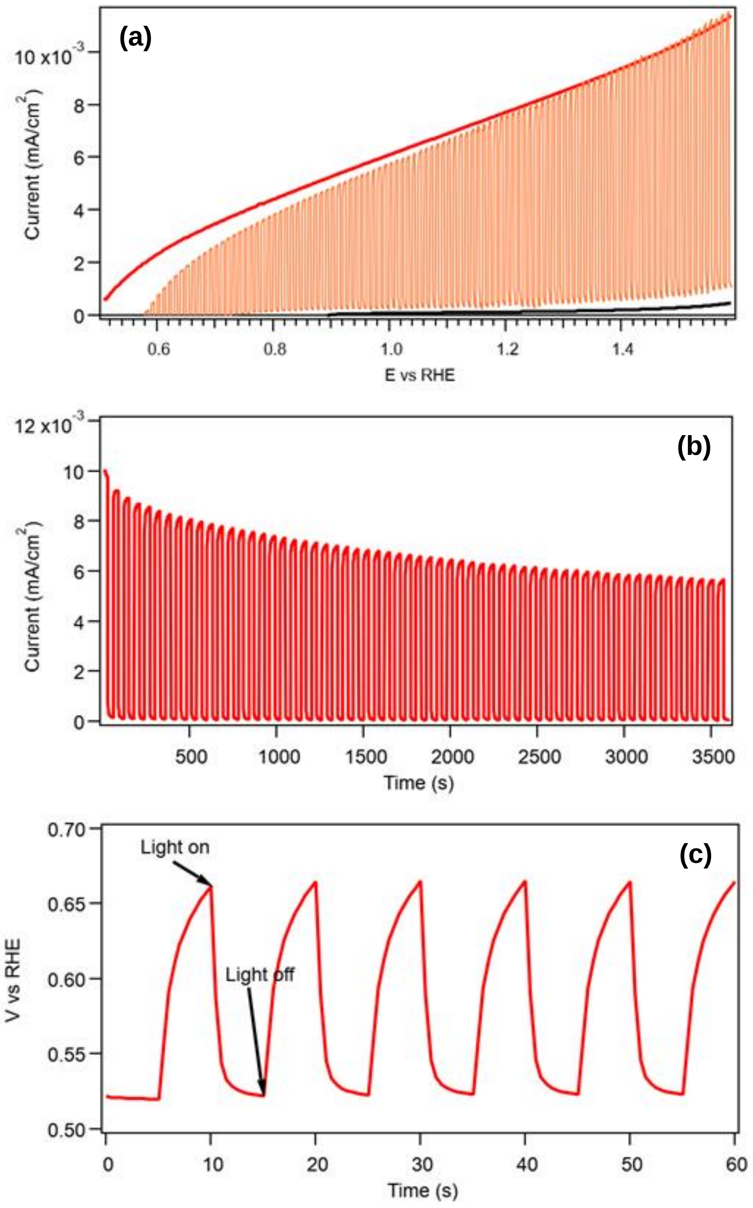

Figure S11. (a) LSV curves in the dark (black curve), under illumination (red curve), and with chopped light (orange curve) for an FTO-supported sample grown at 550°C from 200 mg of melamine; (b) chronoamperometric curve at 1.23 V vs. RHE under chopped light for the same sample; (c) OCP scan under chopped light. For all experiments, light intensity was set at 55 mW/cm^2.

S5. Chemico-physical characterization after electrochemical tests

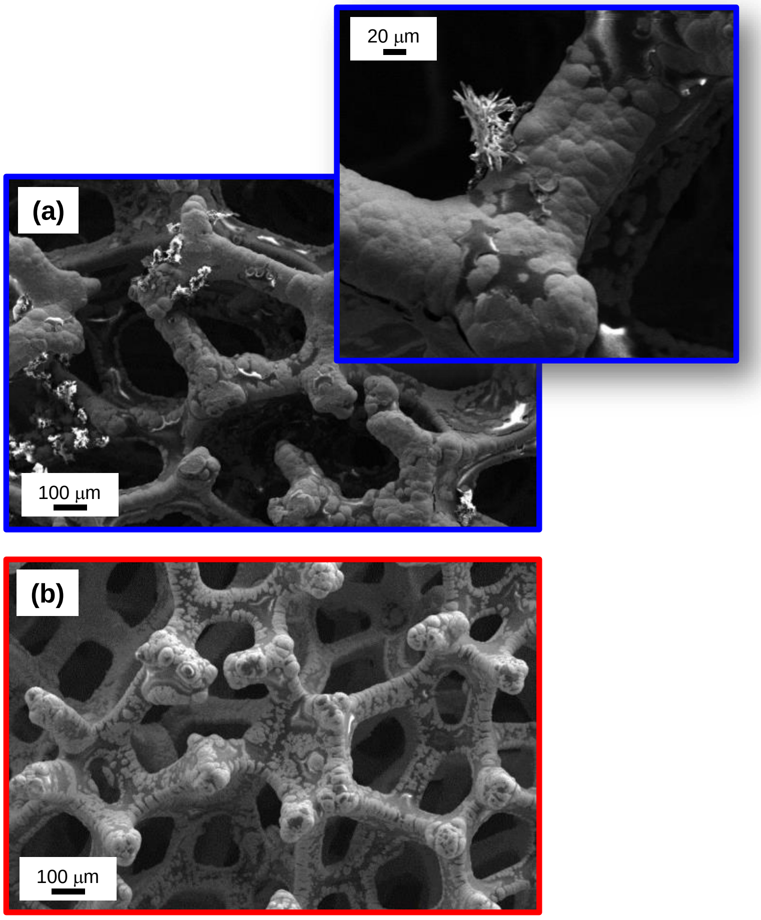

Figure S12. FE-SEM micrographs obtained collecting the SE signal and recorded after electrochemical tests on two samples grown from 300 mg of melamine at (a) 550°C and (b) 500°C.

References

1. Lau, V.W.-h.; Mesch, M.B.; Duppel, V.; Blum, V.; Senker, J.; Lotsch, B.V. Low-molecular-weight carbon nitrides for solar hydrogen evolution. *J. Am. Chem. Soc.* **2015**, *137*, 1064-1072, doi:10.1021/ja511802c.
2. Lau, V.W.-h.; Lotsch, B.V. A tour-guide through carbon nitride-land: structure- and dimensionality-dependent properties for photo(electro)chemical energy conversion and storage. *Adv. Energy Mater.* **2022**, *12*, 2101078, doi:https://doi.org/10.1002/aenm.202101078.
3. Peck, M.A.; Langell, M.A. Comparison of nanoscaled and bulk NiO structural and environmental characteristics by XRD, XAFS, and XPS. *Chem. Mater.* **2012**, *24*, 4483-4490, doi:10.1021/cm300739y.
4. http://srdata.nist.gov/xps.
5. Moulder, J.F.; Stickle, W.F.; Sobol, P.E.; Bomben, K.D. *Handbook of X-ray photoelectron spectroscopy*; Perkin Elmer Corporation, Eden Prairie, MN, USA: 1992.
6. Ohn, S.; Kim, S.Y.; Mun, S.K.; Oh, J.; Sa, Y.J.; Park, S.; Joo, S.H.; Kwon, S.J.; Park, S. Molecularly dispersed nickel-containing species on the carbon nitride network as electrocatalysts for the oxygen evolution reaction. *Carbon* **2017**, *124*, 180-187, doi:https://doi.org/10.1016/j.carbon.2017.08.039.

7. Guo, B.; Tian, L.; Xie, W.; Batool, A.; Xie, G.; Xiang, Q.; Jan, S.U.; Boddula, R.; Gong, J.R. Vertically aligned porous organic semiconductor nanorod array photoanodes for efficient charge utilization. *Nano Lett.* **2018**, *18*, 5954-5960, doi:10.1021/acs.nanolett.8b02740.
8. Chen, Z.; Wang, H.; Xu, J.; Liu, J. Surface engineering of carbon nitride electrode by molecular cobalt species and their photoelectrochemical application. *Chem. Asian J.* **2018**, *13*, 1539-1543, doi:https://doi.org/10.1002/asia.201800487.
9. Benedet, M.; Rizzi, G.A.; Gasparotto, A.; Lebedev, O.I.; Girardi, L.; Maccato, C.; Barreca, D. Tailoring oxygen evolution performances of carbon nitride systems fabricated by electrophoresis through Ag and Au plasma functionalization. *Chem. Eng. J.* **2022**, *448*, 137645, doi:https://doi.org/10.1016/j.cej.2022.137645.
10. Sima, M.; Vasile, E.; Sima, A.; Preda, N.; Logofatu, C. Graphitic carbon nitride based photoanodes prepared by spray coating method. *Int. J. Hydrogen Energy* **2019**, *44*, 24430-24440, doi:https://doi.org/10.1016/j.ijhydene.2019.07.243.
11. Yan, J.; Wu, H.; Chen, H.; Pang, L.; Zhang, Y.; Jiang, R.; Li, L.; Liu, S. One-pot hydrothermal fabrication of layered β-$Ni(OH)_2$/g-C_3N_4 nanohybrids for enhanced photocatalytic water splitting. *Appl. Catal., B* **2016**, *194*, 74-83, doi:https://doi.org/10.1016/j.apcatb.2016.04.048.
12. Bian, J.; Li, Q.; Huang, C.; Li, J.; Guo, Y.; Zaw, M.; Zhang, R.-Q. Thermal vapor condensation of uniform graphitic carbon nitride films with remarkable photocurrent density for photoelectrochemical applications. *Nano Energy* **2015**, *15*, 353-361, doi:https://doi.org/10.1016/j.nanoen.2015.04.012.
13. Xie, X.; Fan, X.; Huang, X.; Wang, T.; He, J. In situ growth of graphitic carbon nitride films on transparent conducting substrates via a solvothermal route for photoelectrochemical performance. *RSC Adv.* **2016**, *6*, 9916-9922, doi:10.1039/c5ra21228f.
14. Jia, Q.; Zhang, S.; Gao, Z.; Yang, P.; Gu, Q. In situ growth of triazine–heptazine based carbon nitride film for efficient (photo)electrochemical performance. *Catal. Sci. Technol.* **2019**, *9*, 425-435, doi:10.1039/c8cy02105h.
15. Ye, L.; Chen, S. Fabrication and high visible-light-driven photocurrent response of g-C_3N_4 film: the role of thiourea. *Appl. Surf. Sci.* **2016**, *389*, 1076-1083, doi:https://doi.org/10.1016/j.apsusc.2016.08.038.
16. Lu, X.; Liu, Z.; Li, J.; Zhang, J.; Guo, Z. Novel framework g-C_3N_4 film as efficient photoanode for photoelectrochemical water splitting. *Appl. Catal. B* **2017**, *209*, 657-662, doi:https://doi.org/10.1016/j.apcatb.2017.03.030.
17. Lv, X.; Cao, M.; Shi, W.; Wang, M.; Shen, Y. A new strategy of preparing uniform graphitic carbon nitride films for photoelectrochemical application. *Carbon* **2017**, *117*, 343-350, doi:https://doi.org/10.1016/j.carbon.2017.02.096.
18. Mohamed, N.A.; Safaei, J.; Ismail, A.F.; Mohamad Noh, M.F.; Arzaee, N.A.; Mansor, N.N.; Ibrahim, M.A.; Ludin, N.A.; Sagu, J.S.; Mat Teridi, M.A. Fabrication of exfoliated graphitic carbon nitride, (g-C_3N_4) thin film by methanolic dispersion. *J. Alloys Compd.* **2020**, *818*, 152916, doi:https://doi.org/10.1016/j.jallcom.2019.152916.
